# “Do it for Your Kid”: Resilience and Mothering in the Context of Intimate Partner Violence in Rural Ontario

**DOI:** 10.34763/jmotherandchild.20242801.d-24-00001

**Published:** 2024-06-19

**Authors:** Kimberley T. Jackson, Panagiota Tryphonopolous, Julia Y. Yates, Katie J. Shillington, Tara Mantler

**Affiliations:** Arthur Labatt Family School of Nursing, Faculty of Health Sciences, The University of Western Ontario, London, Ontario, Canada; Health and Rehabilitation Sciences, Faculty of Health Sciences, The University of Western Ontario, London, Ontario, Canada; School of Health Studies, Faculty of Health Sciences, The University of Western Ontario, London, Ontario, Canada

**Keywords:** resilience, gender-based violence, mothering, rural, women, intimate partner violence

## Abstract

Intimate partner violence (IPV) includes multiple forms of harm inflicted on an intimate partner. Experiences of IPV impact mental and physical health, social relationships, and parenting and resilience may play an important role in how women overcome these detrimental effects. There is little research on how resilience relates to mothers' experience of IPV. We explored the role of resilience in the context of mothers who have experienced IPV in rural settings via semi-structured interviews with six women and 12 service providers. The relationship between resilience and motherhood was a common theme across all narratives. From this theme emerged three subthemes: 1) breaking the cycle of abuse; 2) giving children the “best life”; and 3) to stay or to leave: deciding “for the kids”. Findings underscore the importance of supporting rural women who experience violence in cultivating their resilience and consideration of policy changes which support trauma- and violence-informed care.

## Introduction

Gender-based violence (GBV), defined as any type of abuse, assault, or harassment that can be linked to dominant societal gender norms, is a complex and pervasive human rights concern (Ottawa Coalition to End Violence Against Women [OCTEVAW], 2022). Intimate partner violence (IPV) is one highly prevalent form of violence situated under the umbrella of GBV and includes the multiple forms of harm inflicted on an intimate partner (Government of Canada, 2022). Understood as a pattern of physical, sexual, or emotional violence perpetrated by an intimate partner in the context of coercive control (Tjaden & Thoennes, 2000), it is estimated that IPV is experienced by up to 44% of women in Canada and globally (Flury et al., 2010; Government of Canada, 2022; World Health Organization, 2013). The adverse effects resulting from IPV are far-reaching and often interconnected, impacting mental and physical health, social relationships, economic well-being, and parenting (Adams et al., 2012; Campbell, 2002; Campbell et al., 2013; Crowne et al., 2011; Eckhardt et al., 2013; Wuest et al., 2003). Intimate partner violence can result in femicide – the murder of a woman – with more than 35% of all murders of women globally committed by an intimate partner (Stöckl et al., 2013). The ubiquitous societal and health effects of IPV position this form of GBV as a significant public health concern.

While IPV occurs across all social, cultural, and economic strata, certain populations are more likely to experience IPV (Davies et al., 2009; Edwards, 2015; Logan et al., 2005; Logan et al., 2007; McFarlane et al., 1999). For example, geographic location – particularly living within rural settings – can lead to an increased risk of experiencing IPV, exacerbate the severity of IPV, and amplify the consequences of IPV. Women who experience IPV often seek support from various services, and in many cases, these services protect women from abuse and the effects of such abuse (Sabina & Tindale, 2008). Furthermore, the literature suggests that while important, service use by women experiencing IPV is complex and shaped by many factors. However, it is clear that in Canada and other countries (e.g., the U.S. and Australia), more severe forms of abuse are associated with higher service use (Ford-Gilboe et al., 2015). Unfortunately, support and service use are often restricted for women living rurally, and the factors implicated are structural in nature. According to a recent critical review of the literature including 63 studies, in rural settings IPV perpetrators tended to enact more chronic and severe forms of IPV compared to urban counterparts (Edwards, 2015). Unsurprisingly, rural women experiencing IPV may suffer poorer psychosocial and physical health consequences than urban women (Averill et al., 2007; Grama, 2000; Pruitt, 2008; Sandberg, 2013). Rural communities often lack the same availability, quality, and accessibility to the IPV resources found in urban settings (Fitzsimons et al., 2011; Sandberg, 2013). Factors common to rurality (i.e., isolation, patriarchal family structures, religiosity, privacy norms, lack of anonymity, substance use, and lack of resources) may result in higher rates of IPV, poorer outcomes, and impaired community responsiveness (Edwards, 2015). For example, research conducted in Canada by Mantler et al. (2021) reported examples of structural barriers to support in rural communities, including limited transportation, shortage of service providers, and scarcity of primary healthcare providers. These findings underscore the importance of geographical location in the context of IPV.

While much of the IPV literature focuses on adverse outcomes, a small body of literature explores resilience or the factors related to resilience among women who experience IPV (Howell et al., 2018). Stemming from the research of women who previously experienced IPV but did not have longstanding emotional/psychological challenges (Anderson et al., 2012), understanding the factors contributing to resilience may illuminate the complex, interrelated factors that allow women to recover and thrive in the face of IPV. From a social-ecological perspective, resilience is conceptualised as the capacity an individual has to sustain their well-being through navigating resources in the face of adversity (Ungar, 2013). Resilience can thus be demonstrated in a multitude of ways, including ‘bouncing back’ and recovering from a stressful event (Smith et al., 2008) to achieving growth after experiencing trauma (Tedeschi & Calhoun, 2004). As such, resilience may play an important role in how women overcome or avoid the detrimental effects of IPV and emerge strengthened despite potentially traumatic experiences. While there is little research on the factors that strengthen resilience among women who experience IPV, there is even less known about how resilience relates to mothers' experience of IPV. One study identified that women experiencing IPV who had children were more likely to demonstrate resilience when they were motivated to end the cycle of violence for the sake of their offspring (Brosi et al., 2020). A U.S. qualitative study by Rollero and Speranza (2020) explored the factors that may strengthen resilience among abused mothers and pregnant women in assisted living. This work highlighted that women's relationships with their children were important with respect to fostering resilience. For example, key narratives discussed how hope for the future and dreaming of a peaceful family life for themselves and their children was crucial for strengthening resilience.

While several theories of resilience exist, and some continuing debate about its definition and uses (VanBreda, 2018), when critically applied, it can have usefulness in understanding the human experience. Family resilience theory is particularly relevant to rural mothers' experience of IVP. Defined as “characteristics, dimensions, and properties of families to be resistant to disruption in the face of change and adaptive in the face of crisis situations” (McCubbin & McCubbin, 1988, p. 247), family resilience theory helps to understand how the context of rurality intersects with and plays a role in how mothers survive and thrive despite their abusive relationships. For example, a 2003 meta-analysis (Walsh, 2003) described how family resilience involves the interplay of nine dynamic processes that help families strengthen ties and bolster and utilise competencies and resources. Among these nine processes, which include socio-cultural, community, family, individual, and biological factors, organisational processes of connectedness, and social and community resources are critical (Walsh, 2003). For example, the mobilisation of economic and social resources, such as seeking financial, social, and other sources of support from the community, are seen as important elements of resilience in the face of adversity. Given the unique challenges of experiencing IPV rurally, such as isolation, lack of community resources, and lack of anonymity (Lanier & Maume, 2009; Annan, 2008), this theory provides impetus for the importance of evaluating the nuance of rural living among mothers who experience IPV. While there is evidence that women who experience IPV demonstrate resilience (Anderson et al., 2012; Humphreys, 2003; Shillington et al., 2022), extant literature derives from women in urban settings. Given the uniqueness of rurality (Crann & Barata, 2016), there is a need to understand how women who have experienced IPV cultivate resilience – the ability to survive, grow and thrive—despite exposure to adversity (Prime et al., 2020; Howell et al., 2018; Munoz et al., 2017). This study addresses this knowledge gap by exploring the role of resilience in the context of mothers who have experienced IPV while living in a rural setting.

## Methods

This cross-sectional, qualitative study was a secondary analysis of a larger study – RISE: Understanding Rural Canadian Women who have Experienced Intimate Partner Violence and the Factors that Shape their Resilience – which explored the factors that contribute to resilience and to rural women's ability to survive, grow and thrive in the context of IPV. This secondary analysis was important to explore the specific and unique experiences of rural mothers who have experienced IPV (please see Mantler et al. (2022) for a full overview of study methods). Using Thorne's (2016) interpretive description approach, which is both constructivist and naturalistic, this pragmatic methodology was appropriate to generate knowledge relevant to both health and social science disciplines.

## Recruitment and Data Collection

Both purposive and snowball sampling were used for participant recruitment. Over 200 study advertisements were posted on Kijiji (a Canadian online platform for exchanging goods and services) targeting rural Ontario communities, and approximately 50 rural women's shelters were provided with recruitment materials. Rural women who had experienced IPV at any time in their lives and service providers were both invited to participate. Interested individuals were asked to contact the research team by email. Individual interviews were conducted with eligible, consenting participants between November 2020 and February 2021. Eligible women needed to live in a rural area of Ontario, have experienced IPV, and have access to a safe computer or telephone, while eligible service providers needed to have worked at an Ontario rural women's shelter for a minimum of six months. One-to-one interviews were initially conducted with 14 women (*n* = 14) and 12 service providers (*n* = 12). Approximately four months later, a second follow-up interview was conducted with six women (*n* = 6) and five (*n* = 5) service providers for the purposes of member checking to ensure accuracy and resonance with participant's experiences. To reduce barriers to participation and to enhance recruitment, women and service providers received a $25 gift card in recognition of their time for the first interview and a $10 gift card for completing a follow-up interview. Among the 14 initial interviews, six women identified as mothers. These data, along with data obtained from all service providers, were analysed for the purposes of the study reported herein.

## Procedures

Ethical approval was obtained from the host institution's Non-Medical Research Ethics Board. One-to-one interviews were conducted with women and service providers, lasting approximately 60 minutes, both initially and at follow-up. For member checking and refining analysis, follow-up interviews were conducted with participants interested in having a second interview. The second follow-up interview was conducted with six women and five service providers, all of whom participated in the initial interviews. Semi-structured questions used for both the initial and follow-up interviews are outlined in Table 1.

**Table 1.** Interview Questions for Women and Service Providers

**Phase of Interview**	**Participant Group**	**Interview Questions**
1	Women (*n* = 14)	What helps to support your resilience? What undermines your resilience? What are some challenges/barriers that you have faced to being resilient?
1	Service Providers (*n* = 12)	What do you think helps to support women's resilience? What do you think undermines women's resilience? What are some challenges/barriers that you have seen women encounter that prevent them from being resilient?
2	Women (*n* = 6)	In your relationship, what made you feel stuck? How did you overcome that feeling of “stuck-ness”? What enabled you to keep moving on when things were difficult? When there were moments of crisis?
2	Service Providers (*n* = 5)	What forces women to stay in their relationships (or keep them “stuck” there)? How do you see women overcome that feeling of “stuck-ness”? What enables women to keep moving on when things are difficult? When there were moments of crisis?

Data collection and analyses were guided by Guba and Lincoln (1989) and Thorne and colleagues' (1997) principles of auditability, fit, dependence, and transferability. Interviews were conducted via Zoom or by telephone (per the participant's preference), with participants having the option to be on or off-screen. All interviews were audio-recorded and transcribed verbatim, and each transcript was anonymised prior to analysis. Field notes were recorded by the researchers conducting the interviews.

## Data Analysis

No participant declined to answer or skip any interview questions apart from demographic questions. Analysis occurred at two intervals, first after initial interviews were completed with both women and service providers and second after all follow-up interviews were completed. Transcribed data were organised using Quirkos qualitative analysis software (Quirkos, 2021). Using Thorne's interpretive description approach to analysis (Thorne, 2016), all 26 transcripts were independently coded by the researchers involved in the interviews and the principal investigator. From this, the research team co-created a preliminary coding structure. Dyads of two researchers were randomly assigned, and each dyad conducted analyses using open and line-by-line coding (Blundell et al., 2020). Dyads then met as a team to refine the coding structure and coding definitions as needed. The researchers utilised memoing to identify theoretical outliers, theorise the relationship and structure of the data, and extract meaning from the data set (Thorne, 2016). This process was repeated until the research team was confident that the coding structure sufficiently covered the data. Once all transcripts were analysed, Quirkos files from each coder were merged, and queries/reports were run on each code and associated data. The team then met to discuss the meaning behind the data and to achieve consensus on findings.

## Results

### Participant Characteristics

The mothers in this study (*n* = 6) were from six different Ontario rural communities, and their population sizes ranged between 2,000 and 21,000. Women ranged from 36–57 years of age (*M* = 40.5) and were diverse with respect to annual household income and employment status, with three working full-time, two unemployed and one self-employed. Annual household incomes in Canadian after-tax dollars ranged from $15,000–$48,000, with only one woman reporting a higher income than the average income of single mothers in Canada ($41,250 CAD) (Statistics Canada, 2023). Half of the women received college-level education, while half received high school-level education. Among the six mother participants, four identified as heterosexual, one as bisexual, and one as “fluid”.

Twelve (*n* = 12) service providers took part in this study, representing eight rural Ontario women's shelters. While women experiencing violence can access any services they deem appropriate to meet their needs (e.g., women's shelters, second-stage housing, and sexual assault centres), the service providers participating in this study were all rural women's shelter staff. These shelters operated throughout various rural Ontario communities and were funded via both the provincial government and charitable donations (all were registered charities). Service providers were an average of 42.2 years of age (ranging from 27 to 59), with all having completed either a college or university education. All service provider participants had worked for their respective employers for at least six months. Service providers had annual household incomes ranging from $32,000-$150,000 CAD (*M* = $89,000 CAD).

### Findings

Overarchingly, the findings from this research – from the perspectives of both the women and service providers – spoke of how being a mother was instrumental in fostering resilience in the context of IPV. Despite the subtle differences in how participants viewed the linkage between having children and being resilient, the relationship between resilience and motherhood was a common theme across participants' narratives. From this overarching theme of motherhood and resilience emerged three subthemes: 1) breaking the cycle of abuse; 2) giving children the “best life”; and 3) to stay or to leave: deciding “for the kids”.

### Motherhood and Resilience

Women and service providers described how being a mother served as either a motivator to draw upon inner strength or was explicitly named a source of inner strength, leading to resilience. One provider stated, “the biggest motivator we see is children” (SP8). For some women, being a mother gave them what they needed to “get up and fight” (N10), whereas for other women, having children motivated them to act strong and in control, even in the face of adversity. One service provider highlighted this, stating:

“Most definitely, their inner strength comes out when they're at their weakest. Like we've seen women all day when they're with their children they have a smile on, they act like nothing is going on. Then as soon as they get their children to bed when they have their time into breakdown, cry, talk to counsellor, connect with them, grieve about all that stuff. But it's amazing when you see a woman come in and all day with her children are awake and she is acting strong and powerful as if there's [nothing] going on”

(SP8).

While the importance of motherhood in fostering resilience was a common thread seen across both women participants and service providers, the ways in which women saw their motherhood affecting their resilience were slightly different for individual participants. Some women perceived their children to be a direct source of inner strength, motivating them to persevere during difficult times – where the child(ren) were felt to *give* the woman strength. For others, women described how they had inner strength and ‘dug deep’ *for* the child(ren). For example, one woman participant described the lengths she would go to protect her children. She expressed that while she did not feel her children gave her inner strength, she did what she felt was needed to protect them because she loved them, even if it meant fighting through challenging situations:

“[My] maternal instinct as a mother, forced me, because if you have those strong maternal instincts, you're going to do everything you can to make sure your kids are ok and not in bad situations, in my opinion. You're going to fight to get them out of it, right? Like you would jump in front of a car or whatever to save them. So, I feel like my natural instincts came in when it came to realise situations and stuff. Like I get up every day because of my kids, so yes, I say it does give me a push, but I wouldn't say it gives me inner strength. It's like I get up to do it because I love my kids. Because I'm a mom and it's a natural instinct for me. Um, inner strength came from me. My kids don't give me strength, I do it because I love them”

(N13).

Similarly, another participant felt that being a mother helped her discover the depths of her inner strength: “I was more concerned about my son's safety, then I was about my- how I was feeling, and then over time that just became a natural instinct, and it had- it no longer was about my son, it was about my inner strength, but I didn't find it until I fought for his protection.”

(N5).

In contrast, some women felt being strong and resilient were directly attributed to her having child(ren), with one woman describing her sources of resilience and inner strength: “Number one would be my son, hands down that's, he's it, he's the reason.”

(N2).

Regardless of whether women conceptualised their inner strength and resilience as being drawn *from* their children or *for* the sake of their children, their motivations appeared to centre around their desire to: 1) *break the cycle of abuse* for their children, and 2) provide their children with the *best life* possible, and, in turn 3) affected their decisions *to stay or to leave* the abusive partner.

#### Break the Cycle of Abuse

Several participants described how they did not want their child(ren) to bear witness to abuse or be a part of an abusive homelife and how their child(ren) motivated them to strive for or create a homelife free from abuse. Service providers explained that for many women, breaking the cycle of violence was an important source of inner strength for mothers. One service provider highlighted this, stating:

“And I also think children, I think, you know, when they have children depending on them, that gives them the strength that, you know, I just cannot sit here and do nothing, I need to, I need to leave, I need to get off I need to do something because my kids are being impacted and I don't want them to continue the cycle of violence”

(SP1).

For several participants, breaking the cycle of violence meant modelling the actions and decision-making that would be required to restore or sustain an abuse-free home life. One participant recounted that breaking the cycle of violence was enacted by showing her daughter what a healthy homelife was like: “My number one goal with raising my children is to raise human beings that are awesome, and I want to hang around, and that will be there when I need them just like I have always done for them. So, I'm going to show them by, well, by doing” (N10). Similarly, another participant described how important it was for her to show her daughter that women can be self-sufficient, and independent, stating:

“I'm doing this, like, for me and my kids. I'm going to show my kids and I'm going to show my daughter, that was a huge one … So you know what she sees mom change light fixtures, she sees mom own her own business. She sees mom do everything herself, like and it's good. It's teaching her that, you know, you don't need a man and that was huge too. I don't want my daughter growing up like this”

(N13).

#### Best Life

Whether it was trying to provide an abuse-free environment or preventing future intergenerational cycles of abuse, a common theme throughout participant and service provider narratives centred on the importance of women providing the “best life” for their children. When women described what gave them inner strength and bolstered their resilience, they articulated a desire to create the best possible life for their children even under adverse circumstances. For one participant, this meant a desire to model a homelife with healthy relationships:

“I just feel like if it wasn't for him, like knowing like you don't want a child to grow up in any such, like any shitty situation, you just don't. You want them to have the best life they possibly can. And just, you want them to see what a healthy relationship is supposed to be like, and what a healthy parent is and not. I don't know, it's so hard to explain”

(N2).

For another participant, providing the best life for her child gave her the strength to ‘stay on track’: “My kids don't give me strength, I do it because I love them and because I want to see them have the best life they can…I'll attribute my children for me staying on track for sure.” (N13). Finally, another participant recalled the importance of providing the best life for her daughter and how this significantly shifted her way of thinking. When asked what contributes to her inner strength, this mother replied, “…definitely the motivation of- of wanting to do the best thing for my daughter, regardless of what that may be. Or regardless of how hard that may be. Wanting to give her the best life possible has definitely been a game changer” (N14). To underscore the relevance of this theme, service providers also reported that they observed women doing whatever they could to provide a better life for their children. A service provider summed up this finding, recounting that women demonstrated resilience as they enacted different ways they were motivated to provide a good, abuse-free life for their family:

“A lot of times if they have children that's a big motivator for them because they want to have a good future for their child … they don't want their child to witness or go through the abuse anymore … that's a big motivator for them”

(SP8).

#### To Stay or to Leave

For women in this study, being a mother played a pivotal role in decision-making around whether or not to leave an abusive relationship. The decision to leave an abusive relationship is multifactorial and complex, but for women in this study, having children added having to consider and weigh out meeting the child(s) basic needs (e.g., income, food, shelter), in addition to physical and emotional safety considerations and wanting what was best for them. In some cases, this meant a woman had to stay in an abusive relationship because she felt it impossible to provide for her child's basic needs by herself. One woman recalled how her in-laws undermined her resilience by calling out her inability to independently provide (financially) for her son: “And they kind of bring my resilience back down, like, ‘well no, you cannot do it without him. You need him. Like, how are you going to be a mom by yourself? How are you going to take care of him? How are you gonna pay the bills?”’ (N2). Similarly, having children played a part in this participant's experience, as she ‘tried to make it work’ in her current relationship: “the fact that we had children together was always a big factor because I was trying to make it work you know, ‘is it really that bad?’ is what I would ask myself” (N10). In contrast, many mothers in this study spoke about how their children were a key motivator to their leaving the abusive partner. To highlight, this participant stated, “Oh, yeah 100 percent for my- yeah. It was literally for my kids, I was like, for myself and my kids, I was like, we're not doing this anymore” (N13).

Service provider participants also spoke of the complexity of decision-making mothers faced concerning whether or not to stay with an abusive partner. This service provider spoke about how women would often stay to maintain the children's home, belongings, and traditional family, but would leave once it was clear the children were unsafe:

“…in the world of supporting women in violence is that they'll do things for their children that they never would do for themselves, right? It's often I left for my kids, I did this for my kids, right? So that's a very common experience that I see almost every time. So I think the kids are initially, again, it's sort of two pronged thing, I think initially, it's what keeps her there, to make sure that there's a roof over their head, there's the traditional family style, there feels like there's more support happening, the kids have their rooms, they have their things. So we stay as long as we can. And then again, when it gets to a spot that it's not able to be tolerated, um they're also the reason why women leave, right? Because often their own sense of self and their own sense of um purpose and even that sense of deserving uh to be safe and have their own needs met, comes later. So I think really it is, it's like a superpower, the way moms can put their children's needs, they'll do things way, way beyond what they think they can do for their kids”

(SP4).

## Discussion

The purpose of this study was to explore the role of resilience in the context of mothers who have experienced intimate partner violence in rural settings. Findings from this study demonstrated that for many participants, being a mother played an instrumental role in her resilience, with children being a source of or reason for finding inner strength. Further, both the perspectives of women and service providers echoed that women would make key decisions, however difficult, based upon what meant meeting their children's needs and/or providing the best life they could for their children.

Experiencing family violence is one of the strongest and most consistent predictors of children becoming abused or abusive in their own adult relationships (Cui et al., 2013; McKinney et al., 2009). For example, witnessing family violence as a child can place girls at a four to sixfold increase in risk for experiencing IPV as an adult (Bensley et al., 2003). Fortunately, supportive and responsive parenting by the nonviolent parent has been shown to promote resilience and thriving among children despite their exposure to IPV (Finkelstein et al., 2005; Graham-Bermann, et al., 2009). The findings from this study align with other literature, which underscores women's desire to promote healthy relationships in their children. For example, a qualitative study aimed at understanding the role mothers play in breaking the cycle of IPV found that women had a desire to help their children avoid violent relationships in their future and felt that this was facilitated through having close and open relationships with their children (Insetta et al., 2014). In addition, our findings align with other IPV literature concluding that mothers who experience IPV are generally nurturing and caring of their children and, in fact, may compensate for IPV exposure by being more sensitive and attentive to the needs of their children (Ford-Gilboe et al., 2005; Letourneau et al., 2007). While the findings from this study and others suggest that women who experience IPV desire to break the cycle of family violence, further research is required to explore this phenomenon more fully.

Numerous modifiable and non-modifiable factors play a role in how women respond to IPV. In contrast to many of these factors, the presence of children is highly complex (Meyer, 2011). Decisions on whether to stay silent, reach out for support, or stay or leave an abusive relationship are situationally dependent and less predictable in the presence of children (Meyer, 2011). Understandably, findings from other studies are inconsistent; where in some cases, having children leads to help-seeking as a means of protecting children (Ellsberg et al., 2001), whereas in others, having children leads to silence for fear of harming or losing children after disclosing abuse (Douglas & Walsh, 2010). The findings from the current study support that maternal decision-making in the context of IPV was highly complex and underscored by the motivation to do what they felt was best for the child(ren). Consistent with other literature, women in this study were faced with the complex decision of staying or leaving an abusive partner. The current study identified that for many women, staying in an abusive relationship where children were involved meant maintaining a traditional family and maintaining the children's home and belongings. This is consistent with extant literature outlining external inhibitory factors which make it difficult for women to leave, such as limited financial resources, threats that the partner will harm the children following a separation, and issues relating to cultural norms and stigmas which pressure women to ‘work things out’ (Cravens et al., 2015). In this study, it is also possible that the issue of rurality further problematised the complexity of decision-making. For example, it is well understood that they have fewer formal supports and have less access to the broad range of support that exists for urban counterparts (Ford-Gilboe et al., 2015; Mantler et al., 2021). Further, in many rural contexts, attitudes toward IPV often undermine health and help-seeking among women experiencing IPV (Mantler et al., 2018). The decision to leave is rarely an isolated event, rather, it is a process that often is triggered by a breaking point in the relationship (Barnett, 2001; Short et al., 2000). Despite the often overwhelming inhibitory factors to leaving reported by women and service providers in this study, most women left the abusive relationship when they feared for the safety or well-being of their child(ren).

### Implications for Practice

Findings from this study underscore the complexity of decision-making around staying or leaving an abusive partner in the context of rural motherhood. These findings may help to inform future directions for policymakers and health and social services providers. Recent research has uncovered the role that maternal identity plays for mothers leaving an abusive relationship, where *awakened maternal identity* (AMI) was seen as a ‘turning point’ leading to women leaving the violent relationship for their children (Secco et al., 2016). As such, community resources and interventions which support maternal identity may assist women in initiating the leaving process safely. Nurses, physicians, allied health care providers and service providers who provide direct support are well-positioned to provide sensitive care to mothers in abusive relationships (Chang et al., 2010). In particular, nurses, nurse practitioners, and family physicians have frequent contact with women and their children and are equipped with the skills to assess bonding, attachment, and other indicators of healthy attainment of maternal identity, which is a precursor to leaving a violent relationship (Secco, 2002).

Providing a health and social service environment where women feel safe, free of judgement, and supported is critical for promoting women's empowerment to make the best choices for their families (Murray et al., 2015). Additionally, some research suggests that care and service providers should be aware of women's ‘turning points’ – such as needs around safety, resources, and support required to facilitate leaving – that inevitably influence a woman's capacity to make the best decisions for her family (Chang et al., 2010). *How* these services are provided can have a massive impact on the health, well-being, and overall effectiveness of such care, whereby a lack of understanding of violence and its sequelae may lead to harm (Wathen et al., 2021). Given the prevalence and ubiquity of gender-based violence, trauma-and violence-informed care (TVIC) approaches are important considerations in contexts where women and mothers are recipients of care. Trauma- and violence-informed approaches requires: 1) fundamental system changes where providers understand trauma and violence and the impact it has on people's lives; 2) the creation of emotionally and physically safe environments; 3) the creation of opportunities for choice, collaboration, and connection; and 4) approaches to care from a strengths and capacity-based perspective (Wathen et al., 2021). There is emerging evidence that the provision of education to health and social service providers about TVIC can lead to positive practice changes (Wathen et al., 2021). As such, consideration should be given to the provision of TVIC training to all health and social service providers dealing directly with mothers, especially in rural, underserviced areas.

### Limitations

Given that this was a secondary analysis and the inherent nature of such analyses, this study is not without limitations. The researchers did not have control over the data collection processes, so the study is limited by the small sample size and the inability to engage in member checking on the findings specific to rural women with children in the context of IPV. While the qualitative nature of this study allowed for in-depth insights into how rural women experiencing IPV foster resilience, the interview questions did not specifically address the issues or factors related to mothering in this context. As such, further research is required to better understand the experiences of rural mothers who experience IPV and how mothering impacts their resilience in the face of IPV. Finally, due to the limited sample size and relative homogeneity of the sample, further research which purposefully includes a more diverse representation of race, culture, and other intersectional variables would be advantageous.

## Conclusion

Intimate partner violence is a pervasive problem, and being a rural mother in the face of IPV may serve to make decision-making even more complex and challenging. However, having children may also serve as a means to motivate, strengthen, and bolster resilience and, in some cases, may be responsible for ultimately leaving the abusive partner. While this research is an important small step in our understanding of this population's experience of IPV and its interplay with motherhood, further research is required to enable health and service providers to better respond to the needs of these women.
